# Sirtuin Proteins and Memory: A Promising Target in Alzheimer’s Disease Therapy?

**DOI:** 10.3390/nu16234088

**Published:** 2024-11-27

**Authors:** Francesca Fernandez, Lyn R. Griffiths, Heidi G. Sutherland, Michael H. Cole, J. Helen Fitton, Pia Winberg, Daniel Schweitzer, Lloyd N. Hopkins, Barbara J. Meyer

**Affiliations:** 1 School of Behavioural and Health Sciences, Faculty of Heath Sciences, Australian Catholic University, Banyo, QLD 4014, Australia; michael.cole@acu.edu.au; 2 Centre for Genomics and Personalised Health, School of Biomedical Sciences, Queensland University of Technology, 60 Musk Ave, Kelvin Grove, QLD 4059, Australia; lyn.griffiths@qut.edu.au (L.R.G.); heidi.sutherland@qut.edu.au (H.G.S.); lloyd.hopkins@hdr.qut.edu.au (L.N.H.); 3 Healthy Brain and Mind Research Centre, Australian Catholic University, Fitzroy, VIC 3065, Australia; 4 Venus Shell Systems Pty Ltd., Huskisson, NSW 2540, Australia; drfiton@rdadvisor.com (J.H.F.); pia@venusshellsystems.com.au (P.W.); 5 School of Medical, Indigenous and Health Science, University of Wollongong, Wollongong, NSW 2522, Australia; bmeyer@uow.edu.au; 6 Mater Centre of Neuroscience, 53 Raymond Terrace, South Brisbane, QLD 4066, Australia; daniel.schweitzer2@mater.org.au; 7 Department of Neurology, Wesley Hospital, 451 Coronation Drive, Auchenflower, QLD 4066, Australia; 8 Molecular Horizons, University of Wollongong, Wollongong, NSW 2522, Australia

**Keywords:** sirtuin, cognition, Alzheimer’s disease, therapeutic agents, natural compounds, exercise

## Abstract

Sirtuins (SIRTs), nicotine adenine dinucleotide (+)-dependent histone deacetylases, have emerged as critical regulators in many signalling pathways involved in a wide range of biological processes. Currently, seven mammalian SIRTs have been characterized and are found across a number of cellular compartments. There has been considerable interest in the role of SIRTs in the brain due to their role in a plethora of metabolic- and age-related diseases, including their involvement in learning and memory function in physiological and pathophysiological conditions. Although cognitive function declines over the course of healthy ageing, neurological disorders including Alzheimer’s disease (AD) can be associated with progressive cognitive impairments. This review aimed to report and integrate recent advances in the understanding of the role of SIRTs in cognitive function and dysfunction in the context of AD. We have also reviewed the use of selective and/or natural SIRT activators as potential therapeutic agents and/or adjuvants for AD.

## 1. Introduction

Research on Sirtuin (SIRT) proteins, a group of histone deacetylases, has significantly grown since the role of silent information regulator 2 (Sir2) proteins was discovered, namely as genetic silencing factors in *Saccharomyces cerevisiae* [1,2,3]. Their role as lifespan modulators has attracted further attention leading to a number of studies of this family of proteins in other organisms. SIRT proteins were subsequently found in humans as a conserved enzyme family of NAD^+^-dependent class III histone deacetylases (HDACs, III) due to their ability to remove the acetyl group from acetylated lysines in histones [4,5]. Seven homologs of SIRT proteins (SIRT1–7) have been reported to have a range of different enzymatic activities and functions [6]. The wide array of acetyl and acyl modifications of cytoplasmic proteins and transcription factors by SIRTs have been found to regulate a range of biological processes including DNA repair, cell survival, metabolism, cardiovascular function, ageing, and molecular and cellular processes involved in the encoding of memory [6,7,8,9,10,11].

**Figure 1:**
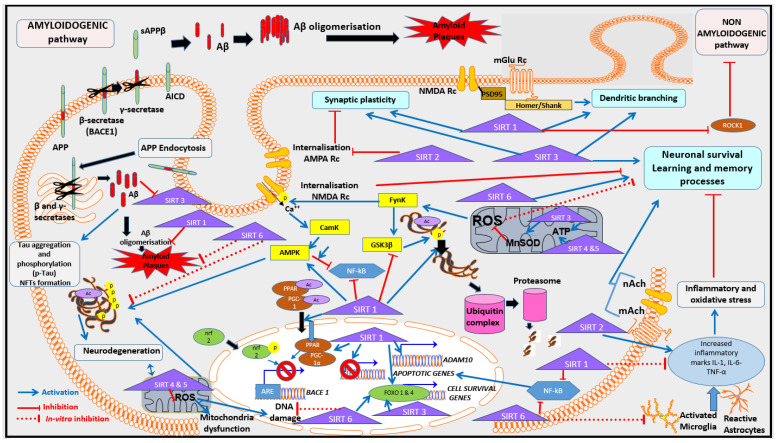
Overview of SIRTs in the context of learning and memory in AD. Briefly, learning and memory process are supported by synaptic plasticity activation and dendrites formation and branching in neurons. SIRT1 and SIRT3 stimulate these processes directly and indirectly via regulation of cellular oxidation, inflammation and degradation of NFTs via ubiquitination. SIRT1 is also involved in the indirect stimulation of the non-amyloidogenic pathway, leading to a decrease in the formation of Aβ in AD. Via its inhibition of AMPA Rc internalisation, SIRT2 promotes synaptic plasticity and memory. SIRT1 participates in the regulation of *BACE1* and *ADAM10* genes, both critical genes in the context of AD. SIRT1, SIRT3 and SIRT6 also modulate the transcription of apoptotic and survival genes in the neurons. Abbreviations: ADAM 10: A Disintegrin and Metalloprotease 10, AMPK: 5′adenosine-monophosphate-activated protein kinase; AMPA Rc: α-amino-3-hydroxy-5-methyl-4-isoxazolepropionic acid receptor, APP: Amyloid precursor protein, sAPPβ: secreted amino-terminals APPβ fragment, BACE 1: β-secretase enzyme, CamK: Ca 2+/calmodulin-dependent protein kinase, cdck5cyclin dependent kinase, Aβ: amyloid-β peptides, AICD: APP Intracellular Cytoplasmic/*C*-terminal Domains, Fynk: Fyn kinase, GSK3β: Glycogen synthase kinase 3 beta, IL-1: Interleukin 1, IL-6: Interleukin 6, mAch and nACh: Metabotropic and nicotinic Acetyl-choline receptors, NF-kB: nuclear factor kappa-light-chain-enhancer of activated B cells, NFTs: Neurofibrillary tangles, NMDA Rc: methyl-D-aspartate receptors receptor, TNF-α: Tumor Necrosis factor alpha, ROS: reactive oxygen species, PSD95: post-synaptic density protein 95.

Over the last decade, there has been increasing evidence to support the critical roles of SIRT in both general health and pathological conditions such as cancer and neurodegenerative diseases. Several randomized controlled trials have demonstrated that SIRT modulators may affect the expression of SIRT proteins in human samples and may have a diverse range of physiological functions, thereby offering a new potential therapeutic avenue for a diverse range of conditions [12,13,14,15]. For instance, SIRT inhibitors have been considered in the treatment of several types of cancer [16], while SIRT activators have been suggested as therapeutic targets for age-related disorders such as Alzheimer’s disease (AD) [17]. In recent decades, traditional medicine has largely focused on targeting the effects of the amyloid cascade, which leads to the proteolytic production of amyloid-beta (Aβ) oligomers from the amyloid precursor protein (APP) (see Figure 1, [18]). As shown in Figure 1, the accumulation of Aβ leads to the formation of amyloid or senile plaques, and an imbalance of the phosphorylation of tau proteins leads to the production of neurofibrillary tangles (NFTs), both hallmarks of AD [18]. The formation and lack of clearance of Aβ oligomers and NFTs contribute to neurodegeneration and progressive neuronal death. Several studies have also emphasized the link between tau pathology and amyloid plaque production, offering substantial evidence for their synergic effects in the AD brain, particularly in the temporal lobe, which is a critical region underlying memory function. Recent studies confirm that targeting specific molecular pathways, such as the cholinergic and glutamatergic systems, holds promise for potentially alleviating AD [19]. However, these treatments have limitations, primarily due to their broad inhibition of normal physiological processes leading to significant adverse effects [19]. In 2021, the newest therapy, Aducanumab, received accelerated approval from the Food and Drug Administration due to its efficacy for reducing AD amyloid plaques [20]. However, this latest method of treatment consists of infusions with monoclonal anti-amyloid antibodies, has a high personal cost (over AUD 50K/year per person), and is only effective in patients with moderate symptoms. In addition, monoclonal antibodies present side effects affecting over 12% of people with AD, requiring medical monitoring for brain oedema, particularly following the first infusion [19]. Consequently, it is essential to continue searching for AD therapies with minimum adverse effects for treating AD patients. For the last decade, natural medicines and/or supplements have attracted interest, and several phytochemical compounds have been shown to reduce AD hallmarks (such as amyloid accumulation, neuroinflammation and oxidation). Interestingly, some of these natural compounds have been reported to act on SIRTs, which for some of the subtypes have been reported at lower levels in AD patients [21].

In this review, we have provided an overview of the different types of SIRT proteins and reviewed their related activity and functional roles in the context of cognitive function and dysfunction. Finally, we have reviewed the recent research advances in the use of selective and/or natural SIRT activators as potential therapeutic agents and/or adjuvants for AD.

## 2. Overview of SIRT Family: Cell Location, Expression and Major Cell Functions

Seven SIRTs have been identified in mammals and reported in subcellular compartments including the cytoplasm (SIRT1 and SIRT2), nucleus (SIRT1, SIRT2, SIRT6, and SIRT7), and mitochondria (SIRT3, SIRT4, and SIRT5) (Table 1) [22]. These seven SIRTs share a common conserved NAD-binding and catalytic core domain, comprising 275 amino-acids, but differ in both *N*- or *C*-terminal extensions [6]. The catalytic site includes a larger domain consisting of a Rossmann folding structure that is typical for NAD^+^ binding proteins and a smaller domain which is formed through the association with a Zn^2+^ binding module and an α helical region representing the highest variability among SIRT family members [23]. Positions of the amino acid residues binding to the Zn^2+^ module vary across the SIRTs, which in turn contribute to the specificity of each member of the family as well as the diversity of their functions [24].

### 2.1. SIRT1

SIRT1 was one of the first SIRT proteins to be discovered, with considerable research highlighting its role in many neuronal processes. Dysfunctional SIRT1-dependent processes are associated with an increased risk of developing pathologies such as Parkinson’s, Hungtinton’s, and Alzheimer’s diseases [25,26,27].

SIRT1 is a NAD-dependent deacetylase that can remove acetyl groups from a large variety of substrates, including histone and nonhistone proteins [28]. SIRT1 is mainly located in the nucleus, but can shuttle to the cytoplasm during neuronal development, differentiation, and in situations where tumor cells are present [29,30,31]. As illustrated in Table 1, SIRT1 plays a key role in terms of modulating a range of cellular functions, including cell cycle and regulation, apoptosis, inflammatory response, oxidative stress, and energy metabolism via the activation of several different cellular pathways. For example, in human/mouse brain cells, SIRT1 has a neuroprotective action via actions on p53 and FOXO (forkhead transcriptional factors FOXO) across several pathways involved in apoptosis [32].

SIRT1 has been reported to have a dual effect on FOXO3 function. When activated, it can increase FOXO3’s (forkhead transcriptional factors, subgroup 3) ability to induce both cell cycle arrest and oxidative stress resistance as well as potentially inhibiting FOXO3’s capacity for inducing cell death [28]. The wide range of roles of SIRT1 has been shown to be important in cardiomyocytes using mouse models where ischemia and hypoxia-induced apoptosis may result in a range of severe cardiac pathologies [33,34]. SIRT1 can also activate FOXO1 (forkhead transcriptional factors, subgroup 1) and FOXO4 (forkhead transcriptional factors, subgroup 4), which are involved in the control of cell proliferation, differentiation, and cell survival [35,36]. The regulation of p53 transcription activity by SIRT1 can either occur directly, via p53 deacetylation, or indirectly, by deacetylating the CBP/p300 acetyltransferase, which can itself acetylate p53 [37,38]. Interestingly, the SIRT1–p53 axis plays a complex role in tumorigenesis, in terms of both promoting and/or suppressing tumor growth [39]. The activation of SIRT1 has been shown to lead to autophagy and cell death in glioma cells and glial malignant cells [40]. Furthermore, SIRT1 upregulation was reported in numerous human cancers, including melanoma, colon, prostrate, breast, liver, lymphoma, leukemia, and sarcomas [41,42,43,44]. SIRT1’s modulation of DNA repair and tumor-suppressing genes, combined with its inhibition of oncogenes and oncoproteins, can lead to it having a protective role against cancer [45,46]. Additionally, recent studies have demonstrated the potential effect of activating the SIRT1–adenosine monophosphate-activated protein kinase (AMPK)/FOXO3 pathway in terms of reversing the chemoresistance and proliferation of cancer stem cells in human gastric cancer tissues [47]. Although the diverse functions of SIRT1 in cancer remain unclear and contradictory, the activation of SIRT1 pathways may lead to a range of beneficial therapeutic effects for cancer.

The role of SIRT1 in the inflammatory response has been widely reported [12,28,48]. SIRT1 is known to modulate inflammation through the deacetylation of histones in the promoter region of inflammatory genes which directly inhibits their transcription. Increased histone 3 deacetylation at lysine 16 (H3K16) may lead to the suppression of genes for inflammatory cytokines such as Interleukin 1 beta (IL-1β) and tumor necrosis factor alpha (TNF-α) [49]. When SIRT1 levels were decreased, the expression of NF-κB p65 was increased, leading to an increase in the expression of inflammatory cytokines TNF-α and Interleukin 6 (IL-6) in hepatocyte cells [50]. Further to its action on histone proteins, SIRT1 can lead to a decrease in the expression of NF-κB-mediated inflammatory cytokines by downregulating the acetylation of P65 (most common activated form of NF-κB) at lysine 310, resulting in anti-inflammatory effects [51]. SIRT1 can also indirectly inhibit NF-κB signaling by its stimulative interaction with AMPK, which is an inhibitor of NF-κB [52].

In summary, SIRT1 possesses a critical role across several important functions in the cell, including the regulation of several crucial proteins implicated in pivotal aspects of cell physiology, such as cell cycle and survival.

### 2.2. SIRT2

In a similar way to SIRT1, SIRT2 is also known to be important in the modulation of cell survival and, as mentioned in Table 1, has been found across both nuclear and cytosolic compartments. The anti-inflammatory role of SIRT2 has been demonstrated when inhibiting SIRT2, thereby leading to the phosphorylation and degradation of NF-κB as well as the reduction in the nuclear translocation of p65 [53]. SIRT2 also plays a critical role in reducing oxidative stress, notably via deacetylation of FOXO and Nrf2 transcription factors [54]. The different roles of SIRT2 in apoptosis, via p53 regulation, as well in cell cycle progression have been reported across several studies which has led to further interest in terms of studying the action of this protein in the context of cancer progression [55,56,57,58]; however, its function as a tumor regulator varies according to the cellular context or the type of tumors present in the cell [57].

In the central nervous system (CNS), SIRT2 is highly expressed in oligodendrocytes, which are glial cells responsible for producing myelin [59]. SIRT2 is also involved in the development of myelination and in its repair, both centrally and in the periphery [60]. Interestingly, reduced myelination occurring in healthy ageing due to a decline in the capacity of oligodendrocyte progenitor cells (OPCs), was improved in aged mice when NAD^+^ was supplemented via β-nicotinamide mononucleotide (β-NMN) to restore SIRT2 nuclear entry in OPCs [61]. Furthermore, the knockout (KO) of *SIRT2* in mice led to altered mitochondrial morphology associated with both an increase in oxidative stress and decreased adenosine triphosphate (ATP) expression [62].

Consequently, SIRT2 plays an essential role in regulating major cell functions, particularly in the context of healthy ageing [63].

### 2.3. SIRT3

SIRT3 is present in both nuclear and cytosolic compartments, like SIRT1 and SIRT2, but is mainly localized to the mitochondria (Table 1) [64]. SIRT3 positively modulates the activity of mitochondria by activating several components of the electron transport chain complexes I and II and acetyl-CoA synthetase [65]. Furthermore, SIRT3 increases the activity of isocitrate dehydrogenase-2 (IDH2) in the tricarboxylic acid (TCA) cycle, and long-chain acyl CoA dehydrogenase (LCAD), which stimulates the β-oxidation of fatty acids (FA), leading to the production of acetyl Co-A and, therefore, enhances cellular respiration [12]. Substrates for SIRT3 also include mitochondrial ribosomal protein L10 [66] and mitochondrial chaperone Hsp10, specifically in response to prolonged fasting [67]. SIRT3 provides protection against oxidative stress (reactive oxygen species, ROS) and activates superoxide dismutase 2 (SOD) during prolonged fasting or calorie restriction [68]. SIRT3 is therefore highly implicated across different areas of mitochondrial metabolism and homeostasis, thereby protecting the mitochondria from damage via a range of mechanisms. Mitochondrial homeostasis is essential for cell survival. If dysfunction occurs in this process, it leads to apoptotic cell death of healthy cells.

An additional SIRT3 substrate is the mitochondrial Lon protease which is one of the major cellular responses to acute stresses that can occur in response to serum starvation, heat shock, oxidative stress, and chronic stress conditions related to prolonged oxidative stress, hypoxia, and ageing [69,70,71,72,73]. SIRT3 deacetylates Lon protease at the L917 site, which is near the catalytic dyad of the protein and can affect its proteolytic activity [74]. Lon protease can degrade damaged or oxidised proteins, such as aconitase (Aco2) and glutaminase C (GLS-1), in response to multiple stresses or metabolic change in the cell [75]. Lon can also regulate mitochondrial (mt) DNA replication and transcription via action on the mt transcription factor A (TFAM) [75]. Decline of mitochondrial capacity, linked with decreased levels of mt RNA and mt DNA and increased oxidative stress, and mitochondrial quality control has been associated with the ageing process [76]. The ability of SIRT3 to regulate the activity of Lon most likely represents one of the important mechanisms that SIRT3 uses to regulate and reprogram mitochondrial functions, including reducing oxidative stress and, potentially, alters its effects across a range of important functions associated with ageing [74,76].

SIRT3 has been associated with longevity in both animal and human studies, with *SIRT3* expression significantly reduced (by approximately 40%) in sedentary older adults compared to younger adults [77]. *SIRT3* KO mice were reported to have significantly shorter lifespans than wild-type mice and spontaneously show an accelerated development of age-related disorders including metabolic syndrome, cardiovascular disease, cancer, and neurodegenerative diseases [78]. When *SIRT3* is deficient, the impairment of mitochondrial respiration and increased ROS production were reported in myoblasts and cancer cells [79]. In cardiomyocytes, *SIRT3* deficiency altered the bioenergetics in mitochondria and caused hyperacetylation of the SIRT3 target optic atrophy 1 (OPA1), leading to an aberrant alignment of trans-mitochondrial cristae and cardiac dysfunction [78]. In the CNS, deacetylation of nicotinamide mononucleotide adenylyltransferase 2 (NMNAT2) has been found to have several neuroprotective properties including its actions on heat shock protein 90 (hsp90) interaction, which result in a refolding of aggregated protein substrates [80]. This finding supports the potentially critical role of SIRT3 in age-related diseases associated with the aggregation of pathological proteins such as α-synuclein in Parkinson’s disease (PD) and amyloid beta (Aβ) in AD [80].

### 2.4. SIRT4

Although SIRT4 has not been as widely studied as SIRT3, SIRT4 is located in the mitochondria like SIRT3, and plays several important roles in cell metabolism and survival [22].

The specific deacetylase activity of SIRT4 on malonyl CoA decarboxylase (MCD) leads to its inhibition, which plays a part in the balance of formation and breakdown of FAs (as a source of energy) [81]. SIRT4 is also involved in regulating the production of ROS in mitochondria due to FA oxidation and the consequential upregulation of activity in the electron transport chain [82].

Additional enzymatic activities of SIRT4 include the targeting of ADP-ribosyltransferase and NAD^+^-dependent deacetylase, reported in pancreatic β cells, and impacting insulin response [83]. SIRT4 is also involved in regulating the production of ROS in mitochondria due to FA oxidation and the consequent upregulation of the activity of the electron transport chain [83]. GDH is critical for glutamine catabolism, which provides energy necessary for the TCA cycle and the synthesis of FAs and amino acids [84]. Subsequently, the decreased ATP/ADP ratio due to inactivation of GDH by SIRT4 in turn leads to decreased insulin secretion [84].

In addition, SIRT4 promotes the catabolism of leucine via the upregulation of methylcrotonyl-CoA carboxylase (MCCC) and inhibits glutaminolysis through GDH modulation [84]. Furthermore, SIRT4 was established as a lipoamidase, and can inhibit pyruvate dehydrogenase (PDH), which also negatively regulates acetyl-CoA production and therefore reduces levels of energy in the cell [84]. Collectively, SIRT4 is a critical modulator of cell metabolism and cellular oxidative stress, significantly affecting cell degeneration.

A few studies have examined and reported the protective role of SIRT4 in the apoptotic process via its influence on the ratio of pro-caspase 9 or 3 to caspase 9 or 3, and on Bax translocation in H9c2 cardiomyoblast cells [85]. Further research is required to further establish the role of SIRT4 in cell-programmed death.

Although SIRT4 may play a protective role in some specific cellular contexts, SIRT4 is proposed to promote mitochondrial fusion, which is one of the hallmarks of age-related deterioration of the mitochondria, by directly interacting with OPA1 [86]. Like many of the other members of SIRT family, SIRT4 has been associated with tumorigenesis [87]; in particular, SIRT4 inhibits the mitochondrial glutamine metabolism in cancer cells, acting as a tumor suppressor [87]. SIRT4 mRNA levels were reportedly reduced across several types of malignant tumor tissues, including those related to lung and bladder cancers [88,89].

**Table 1:** Summary of the effects of known mammalian SIRTs, their localization, activity and main functions.

SIRT	Cell Location	Tissue Expression	Enzyme Activity	Major Functions	Implication in AD Pathophysiology and/or Therapy (In Vitro/In Vivo Animal Model for AD)
SIRT1	Nuclear Cytosolic	Brain, retina, musculo-skeletal tissue, adipose tissue, heart, liver kidney, testis, uterus, blood vessels	Deacetylase, deacylase	DNA repair, Chromatin regulation, Cell cycle, Cell, survival, Neuroprotection, Metabolism, Cardio-vascular protection, Inflammation Ageing.	SIRT1 is involved in the regulation of APP processing and the clearance of Aβ and its activation provides neuroprotection [90]. SIRT1 modulates tau phosphorylation by deacetylating tau protein and influencing the activity of kinases and phosphatases, such as PP2A activation leading to a decrease in tau phosphorylation [91]. SIRT1 regulates neuroinflammation via NF-κB [52,92], highly expressed in AD. SIRT1 promotes mitochondrial biogenesis via activation of mitochondrial PGC-1α gene expression [93], which may lead to a reduction in oxidative stress reported in AD.
SIRT2	Nuclear Cytosolic	Brain, musculo-skeletal tissue, adipose tissue, heart, liver kidney, blood vessels	Deacetylase, deacylase	Cell control, Neuroinflammation, Myelination, Oxidation, Metabolism.	Activation of SIRT2 promotes tau phosphorylation via ERK activation. SIRT2 inhibition leads to reduced tau pathology and an increase in Aβ clearance in AD [94,95,96].
SIRT3	Mitochondrial Nuclear Cytosolic	Brain, musculo-skeletal tissue, adipose tissue (particularly brown), heart, liver kidney, oocytes blood vessels	Deacetylase, Decrotonylase	Cell, survival, Neuroprotection, Metabolism, Mitochondrial homeostasis, Inflammation, Oxidation (including FA), Thermogenesis, Ageing.	SIRT3 activation promotes the increase in neuronal survival and decrease apoptotic gene expression, reduces oxidative stress, regulates mitochondrial homeostasis (including increased bioenergetics), decreases neuroinflammation present in AD [97,98,99]
SIRT4	Mitochondrial	Brain, heart, kidney, liver, blood vessels, pancreatic β-cells	Deacetylase, ADP-ribosyltransferase, Lipoamidase, Deacylase	DNA repair, cell survival, Neurodegeneration, Cardio-vascular protection, Oxidation, Metabolism.	SIRT4 activation decrease oxidative stress and increase energy production in mitochondria [81]. SIRT4 modulates apoptosis via the mTOR pathway in AD [100].
SIRT5	Mitochondrial Nuclear Cytosolic	Brain, heart, kidney, liver, blood vessels, testis, thymus, musculoskeletal tissue	Deacetylase, Desuccinylase, Demalonylase, Deglutarylase	Oxidation (including FA)	SIRT5 regulates mitochondrial enzymes (via post-translational modifications), energy metabolism and response to oxidative sterss (via SOD2) [101]. Activation of SIRT5 represses Aβ production in AD by targeting autophagy [102]
SIRT6	Nuclear Cytosolic	Brain, retina, heart, kidney, liver, blood vessels, musculoskeletal tissue, thymus, testis, ovary	Deacetylase, Demyristoylase, ADP-ribosyl-transferase, Deacylase	DNA repair, Metabolism, Cardio-vascular protection, Inflammation Ageing.	Activation of SIRT6 is provides DNA stability and promotes DNA repair, leading to neuronal protection in AD [103]. Activation of SIRT6 reduces tau phosphorylation by deacetylating of tau protein [104].
SIRT7	Nucleolar Nuclear	Brain, heart, kidney, liver, blood cells, musculoskeletal tissue, spleen, testis	Deacetylase, Desuccinylase	Metabolism, Thermogenesis, Ageing.	SIRT7 deficiency protects against Aβ-induced apoptosis via regulation of ROS in cells [105].

In summary, while playing a similar role in tumorigenesis to other SIRTs, SIRT4 acts in contrast to SIRT3 and SIRT5 by regulating several metabolic pathways, including those related to FA oxidation and glutamine catabolism.

### 2.5. SIRT5

SIRT5 is mainly present in the mitochondria and plays a major role in cell metabolism and oxidation, with a smaller proportion present in the cytosol and the nucleus. Unlike the other mitochondrial SIRTs, SIRT5 has weak deacetylase activity, and its action occurs via the desuccinylation, demalonylation, and deglutarylation of the mitochondrial enzymes essential to metabolic pathways, including glycolysis and FA oxidation, and participates in the regulation of the urea cycle [106,107]. Furthermore, the function of SIRT5 in metabolic control is context-dependent, as SIRT5 can either promote or prevent metabolic enzymes depending on cell type and the availability of nutrients [107].

SIRT5 was identified to primarily demalonylate the enzymes involved in glycolysis and gluconeogenesis, such as glyceraldehyde 3-phosphate dehydrogenase (GAPDH) [108]. It also suppresses the activity of Pyruvate Dehydrogenase Complex (PDC) in the TCA cycle via desuccinylation, leading to a reduction in Acetyl-CoA in the mitochondria [109]. However, *SIRT5* KO in HEK293 cells results in impaired pyruvate-dependent complex I- and complex II-driven respiration, suggesting that the function of SIRT5 in the TCA cycle and glycolysis may be context-dependent [110]. During calorie restriction and/or fasting, SIRT5 enhances FA oxidation via the activation of 3-hydroxy-3-methylglutaryl-CoA synthase 2 (HMGCS2) leading to the production of ketones and increases in LCAD activity [111]. The oxidation of FA by SIRT5 also occurs though desuccinylasation and the activation of VLCAD in the mitochondria [112].

SIRT5 downregulates ROS levels in the cell by desuccinylating isocitrate dehydrogenase (IDH2), which activates this enzyme [109]. Overall, similar to the actions of SIRT3, SIRT5 protects cells from oxidative stress, while SIRT4 exacerbates oxidative stress. Over the course of ageing, an imbalance of ROS levels leads to the disturbance of both mitochondrial and cellular homeostasis, which has been related to a decline in SIRT activity.

### 2.6. SIRT6

Along with SIRT3, SIRT6 has been recognised as an important “longevity” protein with a well-established role in ageing processes, although the simplistic view of longevity versus the prevention of accelerated ageing needs to be considered with caution [113].

Although SIRT6 is mainly present in the nucleus, it can also be found in the cytosol. SIRT6 has the capacity to regulate DNA structure and repair and control cell metabolism, leading to a range of anti-ageing effects across a range of cells and subtypes of tissue.

Regulation of chromatin remodeling and DNA repair occurs via SIRT6 deacetylation of histone protein H3 (on lysines K9ac, K56ac and K18ac), which is a critical process for the modulation of gene transcription and chomatin silencing [114,115]. In response to DNA damage, SIRT6 recognizes DNA’s tunnel-like structure and forms a macromolecular complex with DNA-dependent protein kinase to promote DNA double-strand break (DSB) repair [116].

Furthermore, SIRT6 induces deacetylation at H3K9ac and maintains the binding of WRN (Werner syndrome protein) to chromatin at the telomere, providing a resistance to defects related to replication, chromosomal fusion, and premature senescence of the cells [114]. In an earlier study, *SIRT6* KO mice had a lower body weight and significantly shorter lifespan (four weeks) compared to wild-type mice. Of the *SIRT6* KO mice, 80% of the females were still alive at 200 days compared to only 10% of males, suggesting a SIRT6-induced gender-specific regulation of lifespan [117]. In addition to showing a premature ageing phenotype, glucose uptake was also increased in *SIRT6* KO mice compared to control mice, reflecting the metabolic effects of SIRT6, similar to that previously reported in a separate sample of *SIRT6* KO mice in a 129/SvJ/BALB/c background [117,118]. For the more recent of these two studies, glucose dysregulation was associated with an increase in the peripheral levels of glucose transporter GLUT1, as measured in the muscle of the *SIRT6* KO mice [117]. Interestingly, mice with a pancreatic β cell-specific *SIRT6* KO developed glucose intolerance and an impaired insulin secretion, caused by reduced deacetylation of the transcription factor FOXO1 which in turn leads to downregulation of glucose-sensing genes such as *GLUT2* [119].

In addition to the regulation of glucose metabolism, SIRT6 is involved in a number of key processes involved in the regulation of cell lipid metabolism by repressing the synthesis of triglycerides and by promoting mitochondrial FA oxidation and maintaining low levels of low-density lipoprotein cholesterol [120].

Altogether, SIRT6 is a great candidate to target for preventing ageing effects in cells. The overexpression of SIRT6 in male mice has been shown to cause an extended lifespan compared to wild-type mice with significant cardioprotective effects against hypoxia [121,122].

At present, few studies have investigated the potential roles of SIRT6 and SIRT7, suggesting that these may be good candidates for future research concerning the SIRT protein family. Both SIRT6 and SIRT7 mediate apoptosis by regulating p53, with SIRT6 reportedly capable of downregulating and/or upregulating apoptotic processes in several cancers [123].

### 2.7. SIRT7

Although SIRT7 was the last SIRT family protein to be discovered, its role in nucleoli and, in particular, genome stability, is critical for cell survival and function. SIRT7 has been relatively less studied than the other SIRT proteins, but has been found to be a β-NAD+-dependent deacetylase enzyme in the nucleoli that modulates the transcription activity of RNA polymerase I [124]. SIRT7 was found to interact directly and indirectly with other proteins in processes such as chromatin remodeling and ubiquitination, and with proteasome proteins involved in DNA repair and genome stability [125,126,127,128]. Furthermore, SIRT7 has been reported to promote cellular survival following genomic stress through its actions on attenuating DNA damage as well as the p53 response systems [129].

The accumulation of genetic damage and decrease in the availability of NAD+ are reportedly associated with ageing [130,131]. Interestingly, the levels of SIRT7 change across healthy ageing and may change differently across the different tissues, with a significant reduction in SIRT7 across a number of tissue types including skin, lung, and heart [132,133,134].

Increased oxidative damage induced by mitochondrial dysfunction is thought to contribute substantially to biological ageing [135]. SIRT7 can regulate mitochondrial function by deacetylation of GABPβ1 (GA binding protein) residues K69, K340, and K369, which are essential to the responses to physiological challenges including cellular stress, fasting, and ageing [136]. Consequently, a deficiency of SIRT7 may lead to the reduction in cellular energy production, an impairment of the oxidation process, and a significant increase in ROS production which similar to what is observed in the ageing process [65]. Through its actions on regulating mitochondrial ribosomal protein transcription, SIRT7 is involved in the modulation of lipid and glucose metabolism as well as the maintenance of energy cellular homeostasis [124]. Although the exploration of SIRT7 function in cell physiology and pathology is still in its infancy, emerging research that has investigated the modulation of the levels of SIRT7 as well as other SIRTs in the cell is a promising avenue for future studies.

In view of the reported role of SIRTs across different cellular processes involved in ageing, it is important to further define how SIRT may potentially be associated with changes across different cognitive processes. Furthermore, there is mounting evidence from a range of studies that SIRT may decrease with advancing age in physiological and in pathological contexts such as late-onset AD.

## 3. Role of SIRT in Cognition Function and Dysfunction: A Focus on AD

Cognitive capacities including memory, learning, attention, and decision making are important across one’s lifespan. However, these functions are affected over the course of healthy ageing and in the context of neurocognitive disorders [137]. Generally, cognitive functions decline with age and, in some cases, may significantly impact the quality of life of millions of people worldwide [138]. In some cases, these age-related physiological changes in cognition may be further amplified in the setting of AD, contributing to significant cognitive deterioration. The cognitive impairments associated with AD typically worsen with time and are accompanied by the presence of inflammation and oxidation in the brain cells, as well as a progressive increase in amyloid plaques in brain regions involved with memory, such as the hippocampus [18].

All seven members of the SIRT family are present in the brain, and the levels of SIRT1, SIRT3, SIRT5, and SIRT6 have been found to be significantly lower in the hippocampus of AD patients compared to elderly healthy controls [21]. SIRT6 levels were also reduced in the middle temporal gyrus of AD subjects compared to matched healthy adults [139]. Furthermore, genetic associations were reported for both *SIRT2* and *SIRT3* with AD in two independent Caucasian case studies, suggesting a potential role for both of these SIRTs in the pathogenesis of AD [140].

The involvement of SIRTs in AD has also been investigated using a range of mouse and cell culture models. An overview of the role of SIRTs based on these studies is illustrated in Figure 1 and Table 1. Adult *SIRT2* KO mice showed a dysfunction in synaptic plasticity accompanied by impaired learning and memory [141]. Interestingly, the acetylation of lysine residues on the α-amino-3-hydroxy-5-methyl-4-isoxazole propionate receptor (AMPA Rc) reduces the internalization and degradation of this receptor, potentially indicating an important role of this post-translational modification in the modulation of the molecular pathway underlying the synaptic plasticity process (Figure 1) [141]. Axonal degeneration in the spinal cord was observed in middle-aged *SIRT2* KO mice exhibiting locomotor dysfunction [142]. This effect was not present in young *SIRT2* KO mice (3.5 months), implying that there may be an age-dependent role of SIRT2 in locomotion and cognition [142].

Epigenetic regulation of learning and memory may involve modifications of gene expression via histone acetylation [143]. Long-term potentiation (LTP), the molecular process essential for synaptic plasticity and consequently learning and memory, is enhanced by histone acetylation in the hippocampus [144]. Through the deacetylation of histone proteins including H1, SIRT1 can modulate the remodeling of chromatin, significantly impacting gene transcription [145]. *SIRT1* KO mice have been shown to exhibit a significant deficit in both short- and long-term memory, which coincided with a decrease in the expression of synaptic plasticity-related markers (i.e., LTP) in the CA1 hippocampal region, and a significant reduction in dendritic branching and spine density within the hippocampus [146]. Knockdown in the expression of *SIRT1* in mice has been shown to adversely impact spatial memory performance, while anxiety and exploratory behaviours remained unaffected [147]. In the same study, analysis of the proteins in the hippocampus of *SIRT1* knockdown mice showed a significant increase in tau hyperphosphorylation (at serine 396 epitope) and a decrease in hippocampal synaptic proteins, including post-synaptic density 95 (PSD95) and glutamatergic receptor sub-units (NMDAR2B) (see Figure 1) [147].

In further studies, SIRT1 was reported to be involved in the upregulation of α-secretase, a disintegrin, and metalloprotease 10 (ADAM10), as well the downregulation of the phosphorylated form of glycogen synthesis kinase 3 beta (GSK3*β*). Collectively, these changes led to the reduced production of A*β* peptides and tau phosphorylation in the hippocampus of 3 X Transgenic mice Tg (PS1_M146V_, APP_Swe_, tau_P301L_) as animal models for AD [148,149]. Both molecular processes are implicated in ageing and cognitive decline. Furthermore, SIRT1 is reduced with ageing of the microglia through IL-1β upregulation, implying that SIRT1 may play a role in the age-dependent synaptic loss and tau-mediated memory deficits that are seen in mouse models, namely KO mice [150]. When SIRT1 is upregulated, inflammatory factors may be downregulated, leading to a decrease in A*β* production in senescence-accelerated mouse prone-8 (SAMP8) mice, which may potentially enhance a range of cognitive processes including learning and memory [151]. Interestingly, high serum levels of SIRT1 were associated with higher cognitive performance in Parkinson’s disease patients compared to individuals with lower SIRT1 levels; hence, it is likely that SIRT1 plays an important role in memory [152].

When knocking out SIRT3 in mice, aged male and female *SIRT3* KO mice exhibited impaired memory in the Morris water maze compared to wild-type aged mice [97]. This agrees with previous studies involving *SIRT3* KO mice of both genders, which were shown to display poor spatial memory and impairment of LTP process in the hippocampus [153].

Compared with wild-type mice, *SIRT3* KO mice showed a number of impairments in spatial memory, which were also accompanied by reduced dendritic branching and complexity in the hippocampus, particularly in the CA1 sub-region [97]. Fewer branch points and simpler dendritic architecture result in fewer synaptic effects, which is in keeping with the types of cognitive deficits that have been reported in *SIRT3* KO mice. Dendritic branching alteration can in turn affect the formation and stability of the synapses, thereby potentially leading to neurological and cognitive disorders such as AD (see Figure 1) [97]. Recent studies suggest that both SIRT2 and SIRT3 act as key factors in the maintenance of synaptic plasticity under physiological conditions. The inhibition of SIRT2 in mice models leads to an increase in the expression of AMPARs at the cell surface, which is accompanied by significant impairments of hippocampal LTP [141]. SIRT2 acts as an AMPAR deacetylase regulating their internalisation and proteostasis [141]. This finding indicates that SIRT2-mediated regulation of AMPARs is necessary and important for synaptic plasticity (Figure 1).

Furthermore, modulation of the acetylome and activation of several metabolic enzymes (e.g., MnSOD) protects function and redox homeostasis in the mitochondria, which impacts the neuronal survival and synaptic signaling mediating for both short- and long-term memory formation, as well as memory retention, in the hippocampus (Figure 1) [97]. This indicates that mitochondrial SIRTs, particularly SIRT3 which is involved in the modulation of MnSOD, are essential for range of cognitive processes including learning and encoding the hippocampal networks [98].

Nuclear SIRTs, including SIRT6, have also been implicated across a range of modulating learning and the memory process. In *SIRT6* KO mice, the accumulation of hyperphosphorylated and hyperacetylated tau presents in the brain impair learning and memory accompanied by increased DNA damage, and cortical apoptotic cells [154].

In a *SIRT6* conditional KO mouse model, impairment in contextual fear conditioning was observed, suggesting a role of SIRT6 in the formation of contextual memory [155]. Interestingly, SIRT6 was reportedly downregulated in an animal model of the early stages AD, with 5xFAD transgenic mice presenting with memory impairment that worsened with age, potentially due to higher methylation levels [156]. This implies that therapies aimed at SIRT6 or related epigenetic targets after early amyloid deposition may potentially reduce the progression of AD associated neuropathology.

Considering the critical roles of SIRTs in cell physiology and survival, including its role in higher-level functions including cognition, the modulation of SIRTs and/or epigenetic targets may offer new ways of ameliorating cognition decline in various neurological disorders, including AD. Collectively, this area of research is still in its initial phase, but it offers new potential pathways of therapy for AD.

## 4. Natural and Non-Pharmacological Modulation of SIRT Activity: A New Avenue of Therapy for AD?

With their pleiotropic roles within cells, SIRTs play an important part in mediating health and disease. As illustrated in Figure 1, AD pathophysiology includes the sequential cleavage of APP, initiated by the enzyme β secretase (encoded by *BACE*) leading to the production of Aβ (amyloidogenic pathway). In contrast, the activation of α-secretase suppresses Aβ production (non-amyloidogenic pathway) [18]. SIRT1, by deacetylation of transcriptional factors related to secretases and by decreasing Rho-associated coiled-coil-containing protein kinase (ROCK 1), leads to the reduction in Aβ in the brain tissues of AD mice models [157,158]. The inhibition of SIRT2 also leads to the reduction in Aβ in the brain due to the increased levels of APP acetylation in K132 and K134 shifting towards the non-amyloidogenic pathway [94]. Another pathway for reducing Aβ production is the activation of autophagy process by SIRT5 reported in APP/PS1 transgenic mice. Interestingly, the SIRT3 expression was reduced by an increase in Aβ-induced acetylated tau in an ex vivo model of cortical neurons from transgenic mice that carry the human tau protein [99]. Elevated phosphorylation and aggregation of tau are widely considered as AD hallmarks, which involve SIRTs 1, 3, and 6 [12,159]. SIRT6 regulates tau protein stability and phosphorylation through GSK3α/β modulation [160]. SIRT1 overexpression was reported to reduce tau phosphorylation [148], while SIRT1 deletion increases tau phosphorylation [91]. There is a large amount of evidence supporting the dephosphorylation of tau as a therapeutic strategy in AD, and SIRT1 and SIRT6 modulation could target tau this way [91,160].

Considering the significant role of SIRTs in AD pathophysiology, the quest to find SIRT modulators for treating AD symptoms is still in progress. The use of NAD+ precursors, such as nicotinamide riboside and nicotinamide mononucleotide, that can activate SIRTs have been examined in the context of human clinical trials due to their solubility and being orally available [161,162] (Clinicaltrials.gov). However, several clinical trials were terminated due to the lack of specificity of these synthetic SIRT1 activators and their failure to achieve the desired therapeutic effects [90]. The cytotoxicity and multi-target effects of activators of SIRT1 have prompted the need for researchers to explore more effective alternatives for activating SIRT1 pathways [163]. Recently, SRT2104 has emerged as one of the most specific small molecule activators of SIRT1, and was well tolerated in a pilot randomized placebo-controlled double-blind phase I trial (NCT00964340) involving a healthy elderly population [164]. However, future clinical trials are warranted to further explore the efficacy, mechanism of action, and safety in the context of AD. With the lack of studies and/or results for synthetic activators of SIRT in the context of AD, the exploration of natural compounds able to modulate the levels of SIRTs is warranted.

### 4.1. Main Natural Coumpounds Modulating SIRTs in AD

Over the last decade, the antioxidant and anti-inflammatory functions of bioactive compounds have attracted much attention from pharmaceutical companies. Several studies have shown that polyphenols, flavonoids, and polysaccharides are compounds that can modulate SIRT pathways [165], offering new avenues for therapy, particularly in the context of AD.

**Figure 2:**
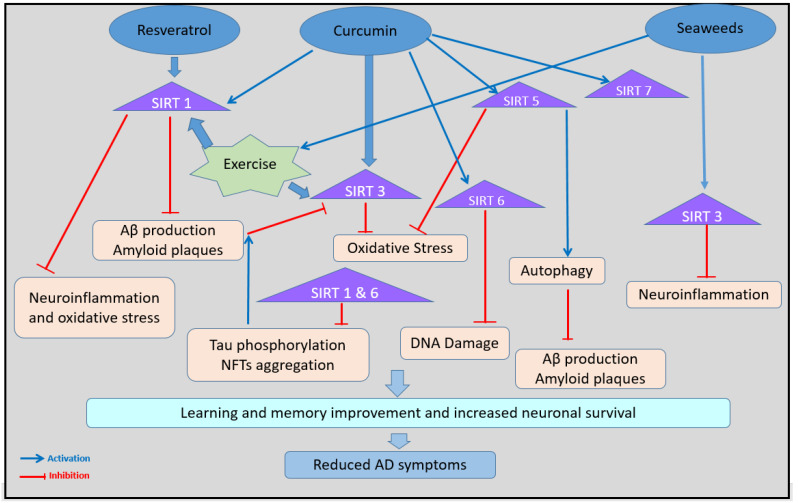
Overview of the modulation of SIRTs by natural compounds (resveratrol, curcumin, and seaweeds) and exercise. Dotted arrows represent an activation of SIRTs reported in vitro.

One of these bioactive compounds tested in AD is resveratrol. Resveratrol can be found in a variety of plants, including grapes, nuts, cocoa, tomatoes, berries, and sugar cane, and is reportedly capable of being able to cross the blood–brain barrier [166]. In the brain, resveratrol possesses the ability to directly bind and activate SIRTs, particularly SIRT1 [167]. Resveratrol regulates SIRT1, and indirectly AMPK, in a dose-dependent manner. At lower levels, resveratrol prompts the activation of SIRT1 to deacetylate liver kinase B1 (LKB1), an upstream kinase of AMPK, leading to an increase in AMPK activity and the elevation of cellular NAD+ levels [167]. Conversely, high levels of resveratrol inhibit ATP production in the mitochondria, which activates AMPK and leads to mTOR inhibition, autophagy, and mitochondrial biogenesis. Meanwhile, SIRT1 activation inhibits the nuclear factor of the kappa-light-chain enhancer of activated B cells (NF-κB), resulting in anti-inflammatory effects (Figure 2) [167]. Interestingly, the autophagy process increases the risk of developing neurological disorders, including AD [168]. Furthermore, resveratrol can block phosphodiesterase (PDE) and cause cAMP and Ca^2+^ levels to rise in the cell, leading to an increase in both AMPK and SIRT1 activities and greater expression of *Nrf2*, in turn regulating the expression of antioxidant genes [169]. These processes may contribute to the neuroprotective (by reducing neuroinflammation and neuronal oxidative stress) and anti-amyloidogenic effects of resveratrol reported in vitro and in vivo, particularly in animal models of AD [170,171,172]. In fact, careful review of the meta-analysis that examined the effects of resveratrol in both human and animal AD cases revealed significant evidence that this compound may be used as a therapeutic and/or protective agent for AD [171]. In a randomised, placebo-controlled, double-blind, multicenter 52-week phase 2 trial of resveratrol, a reduction in CSF Aβ40, Aβ42, and serum Aβ40 was observed in AD individuals receiving resveratrol compared to placebo [170,173]. Although the mechanism via which resveratrol was able to reduce Aβ plaques in the brain remains unclear, this compound may regulate APP processing towards the non-amyloidogenic pathways, leading to reduced production of Aβ peptides [18] (Figure 1 and Figure 2). In addition, resveratrol-induced SIRT1 activation can enhance the ability of astrocytes to clear Aβ plaques during the early stages of disease, consequently delaying the formation of amyloid deposits in the brain [174].

SIRT1 activation by resveratrol also induces glial activation and contributes to increased neurogenesis in the hippocampus via Wnt signaling, which in turn may potentially help improve and restore memory impairment in AD [175]. Previous studies investigating individuals with mild to moderate AD who were administered with resveratrol showed enhanced brain volume, better Mini Mental State Examination (MMSE) scores, and improved scores on tests including the ADAS-cog (AD assessment scale for cognition) compared to control groups (Figure 2) [176,177,178]. These findings should be considered with caution, however, as some studies have reported no significant effects of resveratrol administration [179,180]. This may be due to variations in study design, participant inclusion criteria, dosage and treatment durations, and overlooking other potentially confounding factors (e.g., medications, co-existing medical conditions, or genetic variations for compound metabolism). Further research is needed to fully understand the therapeutic effects of resveratrol in human studies.

Variations in the bioavailability of natural bioactive molecules may also affect the efficiency and therapeutic effects of tested compounds in humans. For example, pharmacokinetic studies of resveratrol showed that this compound was rapidly metabolized and highly absorbed in humans; however, only 2% of free resveratrol was detected in participants’ blood [181]. Nanosized delivery systems were therefore investigated to increase CNS permeability in the context of AD. By encapsulating resveratrol in polymer nanoparticles with a controlled size (1–100 nm), the efficiency of this compound was significantly increased [172]. The natural bioactive compound, curcumin, has shown effectiveness in AD treatment in human randomized trials due to its anti-inflammatory and antioxidant effects [182]. Due to its rapid systemic elimination, the delivery of curcumin via nanoparticles was used to increase curcumin circulation time in the body, and improve its reach to critical regions of the brain [183,184].

Interestingly, nanoparticles encapsulating curcumin can result in a reduction in in vitro and in vivo A*β* generation and aggregation, as reported in postmortem brain tissue from AD patients, and transgenic mice overexpressing AD-related human mutations (APPxPS1 mice) [185]. Curcumin is a hydrophobic polyphenol extracted from the rhizomes of *Curcuma longa*, also known as turmeric, and has been used extensively in Ayurveda Indian and Chinese Medicine for centuries in the management of various diseases, mainly for its anti-inflammatory properties [186]. Its anti-inflammatory activity can be attributed to the inhibition of cyclooxygenase-2 (COX-2) and nitric oxide synthase (NOS) enzymes via the downregulation of NF-κB and cytokines (TNFα and interleukins) [187]. Interestingly, curcumin increases the levels of superoxide and AMPK, and decreases the level of ATP in vascular smooth muscle cells, leading to the upregulation of all tested SIRTs (i.e., SIRT1, SIRT3, SIRT5, SIRT6 and SIRT7) (see Figure 2) [188]. This may explain the improved spatial memory observed in animal models of AD treated with curcumin when compared to sham control groups in various pre-clinical studies (Figure 2) [186,189,190,191,192]. Improved memory was also accompanied by the suppression of neuronal apoptosis in the hippocampus due to the management of apoptotic gene expression, mainly driven by *bax* and *bcl2* [193]. A recent randomised, double-blinded, placebo-controlled pilot trial, involving only 16 participants per group, reported that the Curcumin–galactomannan complex (CGM), administrated for 24 weeks, significantly improved serum inflammatory markers (TNFα and IL6) and MMSE scores [194]. Its delivery, via self-emulsifying hydrogel developed by the uniform encapsulation of curcumin within a soluble dietary fiber hydrogel matrix, was stated to possess an enhanced bioavailability of free (unconjugated) curcuminoids, leading potentially to an improved blood–brain barrier permeability [194]. Larger-scale human trials are required to further establish the efficiency of CGM encapsulated in the hydrogel matrix, within the context of AD.

Despite the positive impact of curcumin across the different cognitive domains, the main challenge in terms of the administration of curcumin in humans is its low bioavailability, due to its rapid first-pass metabolism, low absorption, and poor blood–brain barrier penetration [189]. In a similar way to resveratrol, formulations of curcumin therapies using nanoparticles, aiming to improve the bioavailability and stability, have been developed for preclinical studies, but are yet to be effective in AD clinical studies.

There have been several studies over recent decades that have specifically investigated the beneficial effects of consuming seaweeds. Seaweeds contain many bioactive compounds, including polyphenols, polysaccharides, fucosterol, and fucoxantin, and have been implicated in possessing several biological activities, including anti-inflammatory and antioxidant effects [195]. However, in most cases, their mechanisms of action are yet to be discovered. Brown macro-algal species, specifically in the *Fucaceae* and *Cystoseira* families, are rich in phenolic molecules and polysaccharides, such as fucoidan [195]. Fucoidan can improve cholinergic activity in the neurons of AD model mice, leading to improved memory performance [196]. Interestingly, the administration of fucoidan has been shown to lead to the activation of SIRT3 in neurons, preventing p53-induced mitochondrial dysfunction and neuronal damage in a deacetylase-activity-dependent manner as reported in AD (Figure 2) [197]. Furthermore, fucoidan improved antioxidant activity both in vitro and in vivo by activating superoxide dismutase and glutathione and stimulating the expression of the anti-inflammatory *Nrf2* gene; both activities that are highly relevant in the context of AD [197].

Aside from brown seaweed compounds, ulvan is a sulfated polysaccharide extracted from green seaweed species [198]. Some compounds isolated from seaweeds can pass through the blood–brain barrier and have been found to have several neuroprotective effects [199]. Like brown algae, ulvan supplements have been reported to possess significant biological properties, such as antioxidant, anti-inflammatory, and anti-hyperlipidemic effects [200]. Interestingly, ulvans can increase levels of SIRT1 in human cell lines [201] and SIRT3 in rodents, with reported improvements in memory performance (Figure 2) [202]. Furthermore, a type of ulvan, known as sulfated xylorhamnoglucuronan, or “SXRG 84” supplement, has been previously shown to decrease levels of inflammation and lipid blood levels in a human double-blind clinical trial setting, with no adverse effects reported by any participants [203]. In view of the potential role of inflammation and lipid metabolism changes in the pathogenesis of AD [204], and the role of SIRTs in regulating lipid metabolism [205], further studies are required to fully explore the therapeutic potential of seaweed in AD.

Despite a renewed interest in alternative medicine to help treat AD through its modulation of SIRTs, there are currently no clinical studies demonstrating the efficiency of tested natural compounds in AD therapy. Improving the bioavailability of natural compounds in humans remains a priority in the development of natural medicine. The modulation of SIRTs via a non-pharmacological approach may help address this challenge.

### 4.2. Non-Pharmacological Modulation of SIRTs: Exercise

There is emerging evidence highlighting the neuroprotective effects of regular exercise in both animal and human studies, with improvements in the level of oxidative stress and brain-derived neurotrophic factor (BDNF) expression, which is critical for synaptic plasticity and cognitive performance [157]. It is proposed that the beneficial effects of exercise, in terms of enhancing brain health as well as reducing the risk of disease, could, in part, be due to the activation of SIRTs. In particular, SIRT1 and SIRT3 are potentially activated, both of which are involved in the modulation of NAD+ levels and biogenesis of mitochondria which in turn leads to a greater availability of energy supply for cells [206].

In an animal model of AD, treadmill exercise inhibited Aβ production in the cortex of an NSE/APPsw-transgenic mouse via an increase in SIRT1 levels and peroxisome proliferator-activated receptor-gamma coactivator 1α (PGC-1α) levels. Collectively, these changes reduced the activity of β-site APP cleaving enzyme (BACE 1) and shifted APP processing toward the non-amyloidogenic pathway [93]. Furthermore, it has been shown that physical exercise significantly alters the NAD^+^/NADH ratio resulting in a greater level of expression of SIRT1 in the brain [207]. More importantly, physical activity reduced memory impairment and synaptic dysfunction in 3 × Transgenic mice via the activation of the SIRT1 signaling pathway (Figure 2) [208].

SIRT3 expression can also be modulated through exercise [206]. An in vitro study reported an upregulation of mitochondrial SIRT3 levels in hippocampal neurons in response to exercise [209]. This outcome was also accompanied by neuronal protection against metabolic and excitotoxic stress [209]. Considering the critical role of the hippocampus in learning and memory, it is not surprising that several studies that have investigated the effects of exercise among individuals with AD have reported positive and beneficial results of the intervention along with significant cognitive improvement [210]. Exercise can reduce Aβ deposition leading to significantly increased synaptic connections in the hippocampal CA1 [211], rescued LTP [212], and improved cognitive performance in animal models of AD [213]. In human studies, acute aerobic exercise of moderate intensity (such as 20 minutes of cycling) has been shown to be beneficial for the cognitive abilities of individuals with mild AD (Figure 2) [214,215].

Altogether, these pre-clinical studies indicate that engaging in regular physical activity, particularly aerobic exercise, may help to delay age-related cognitive impairment in older adults and mitigate the rate of memory decline in people with AD. However, the results of clinical trials involving people with AD are varied, and depend on the type, duration, and intensity of exercise and disease stage [216]. Future cohort studies should consider stratification according to disease severity as well as the use of combined interventions aimed at examining appropriate exercise prescriptions for patients with AD.

Additional work is required to further establish the potential therapeutic benefits of SIRTs modulation in AD patients. A combined approach including a low natural compound supplementation and exercise program may maximise the clinical benefit of SIRT modulation. AD patients may benefit from the synergic effects on SIRT pathways from both interventions aiming to regulate SIRT activity leading to the reduction in AD progression and potential improvements in lifestyle.

Furthermore, individual inter-variability reported in clinical trials may also reflect the need to consider genetic make-up involved in tested compound metabolism (such as resveratrol [217] and/or genetic variations within SIRTs genes [140,218]). By taking into account the genetic profiling, the compound doses and/or type of intervention may be further considered and tailored to the individual to maximise the therapeutic effects of SIRT modulation.

## 5. Conclusions

In this review, we have demonstrated that the pathophysiology of AD is closely associated with SIRTs and their related pathways. Further work aimed at examining the modulation of SIRTs may offer a novel strategy for alleviating AD symptoms and enhancing cognitive dysfunction. With the presence of extensive adverse effects demonstrated in the use of conventional treatment for AD, there is a growing emphasis towards developing alternative avenues of therapy with fewer negative physiological effects [18]. For example, the cholinergic esterase inhibitor, Donepezil, can cause extrapyramidal symptoms, bradycardia, gastrointestinal bleeding, and vomiting, while rivastigmine increases the risk of all-cause mortality, especially among patients who are critically ill [219]. Based on this review, and pre-clinical studies on alternative approaches to treat AD, the mechanism of natural molecules and/or exercise to improve the neurodegeneration of AD via modulation of the SIRTs pathways offers a promising avenue of therapy for this disease. Future research is needed to improve drug delivery pathways (such as nanoencapsulation and/or hydrogel matrix) to increase tissue-targeting concentrations and enable these natural molecules to act on target tissues. Although nanotechnology approaches have improved dramatically in the last decade to enhance the durability, solubility, and transport of the bioactive compounds to the brain, the utilization of this technique is still in its infancy and requires more work for its application in human participants. Furthermore, the study of a tailor-made approach according to the individual genetic and epigenetic profiles should be considered in the context of future AD clinical trials.

In conclusion, the quest for improving efficacy and delivery of natural compounds in AD context must continue considering the growth in prevalence of this disease worldwide. A tailor-made approach in these trials could offer a new avenue of therapy for this devastating pathology and potentially lead to the development of effective and disease-modifying therapy.
